# *Leuconostoc citreum*: A Promising Sourdough Fermenting Starter for Low-Sugar-Content Baked Goods

**DOI:** 10.3390/foods13010096

**Published:** 2023-12-27

**Authors:** Maria-Florina Roșca, Adriana Păucean, Simona Maria Man, Maria Simona Chiș, Carmen R. Pop, Anamaria Pop, Anca C. Fărcaș

**Affiliations:** Faculty of Food Science and Technology, University of Agricultural Sciences and Veterinary, 400372 Cluj-Napoca, Romania; maria-florina.rosca@student.usamvcluj.ro (M.-F.R.); simona.man@usamvcluj.ro (S.M.M.); simona.chis@usamvcluj.ro (M.S.C.); carmen-rodica.pop@usamvcluj.ro (C.R.P.); anamaria.pop@usamvcluj.ro (A.P.); anca.farcas@usamvcluj.ro (A.C.F.)

**Keywords:** lactic acid bacteria, *Leuconostoc citreum*, sourdough, sugar reduction, baked goods

## Abstract

This review highlights *Leuconostoc citreum*’s promising possibilities as a proficient mannitol producer and its potential implications for sugar reduction, with a focus on its use in sourdough-based baked good products. Mannitol, a naturally occurring sugar alcohol, has gained popularity in food items due to its low calorie content and unique beneficial qualities. This study summarizes recent research findings and investigates the metabolic pathways and culture conditions that favor increased mannitol production by *Leuconostoc citreum*. Furthermore, it investigates the several applications of mannitol in baked goods, such as its function in increasing texture, flavor and shelf life while lowering the sugar content. Sourdough-based products provide an attractive niche for mannitol integration, as customer demand for healthier and reduced-sugar options increases.

## 1. Introduction

### 1.1. Leuconostoc—History, Morphology and General Importance

The name *Leuconostoc* is a combination of the Greek word *leucus*, meaning clear, and the generic Latin noun *nostoc*, which refers to an alga [1] (*Leuconostoc*, colorless nostoc) [2].

*Leuconostoc* spp. are Gram-positive lactic acid bacteria (LAB) that are significant for the economy. Different environmental *Leuconostoc* strains naturally develop from greenery and roots, which act as their ecological habitat [3], and they spread to diverse niches such as raw milk or cold food goods [2]. Their population is often small, making up less than 1% of the overall microbial community [3]. 

*Leuconostoc* strains, which belong to the low G and C branch of the Gram-positive bacteria, are non-motile, non-spore-forming cocci that are typically seen in pairs or short chains. They lack catalase and arginine dehydrolase and are heterofermentative, creating CO_2_ from the metabolism of glucose in addition to d-lactate, ethanol or acetate. They are mesophilic facultative anaerobic bacteria that grow at 10 °C and exhibit mesophilic features [3].

Farrow et al. (1989) [4] classified *Leuconostoc citreum* as a lactic acid bacteria (LAB) species. It was recognized as a novel species by DNA similarity with other *Leuconostoc* spp. strains but is not one of the original species of the genus *Leuconostoc*. *Leuconostoc citreum* has been isolated from a variety of plant-based food and beverage fermentations including kimchi [5], sourdough [6] and fermented corn beverages [7]. They accomplished a taxonomic distinction between *Leuconostoc citreum* and other *Leuconostoc* species by using primarily DNA-related evidence, which led to the reclassification of strains formerly categorized under *Leuconostoc mesenteroides* [4]. Spherical or lenticular cells (cca. 0.5 to 0.7 by 0.7 to 1.2 pm) are seen in pairs or short chains in *Leuconostoc citreum*. The majority of strains generate a lemon-yellow color and grow between 10 °C and 30 °C; certain strains grow at 37 °C, but when the temperature reaches 40 °C they stop growing. Because it cannot generate acid from ribose, *Leuconostoc citreum* is easily distinguished from *Leuconostoc mesenteroides* subsp. *mesenteroides* and *Leuconostoc pseudomesenteroides*. *Leuconostoc citreum* strains are also negative for α- and β-D-galactosidase and β-D-xylosidase, but *Leuconostoc mesenteroides* subsp. *mesenteroides* and *Leuconostoc pseudomesenteroides* are positive. Another distinctive feature is the capacity to use citrate. The ability of *Leuconostoc citreum* to metabolize citrate is reflected in its name (“*citreum*” refers to citrate) [4]. 

*Leuconostoc* spp. are of tremendous industrial importance since they serve as novel starter cultures for fermented foods (fermented vegetables, dairy products, meats and fresh alcoholic drinks) as well as host strains to develop value-added bioproducts in the field of biotechnology [8]. *Leuconostoc* can be detected in mixed-strain cultures at mesophilic temperatures but are notably absent in starter cultures that operate under thermophilic conditions. The use of starter cultures, including *Leuconostoc citreum,* ensures consistent and reliable results [9].

*Leuconostoc citreum* is a heterofermentative lactic acid bacterium (LAB) that creates lactic acid, acetic acid, alcohol, mannitol, CO_2_ and aromatic compounds [10]. Additionally, genes for the production of dextran polysaccharide from sucrose were reported for *Leuconostoc citreum* EFEL2700 [11], while for *Leuconsotoc citreum* SK24.002 an exopolysaccharide with high resistance for dextranase was found [12]. 

Fermentation can enhance food’s nutritional quality by improving digestibility and increasing the levels of vitamins and minerals compared to the initial content. This process also generates antimicrobial compounds like organic acids, hydrogen peroxide, diacetyls and bacteriocins, which both inhibit undesirable bacterial growth and extend the food’s shelf life. Fermented food products with higher lactic acid content may improve the utilization of calcium, phosphorus and iron, while also increasing the absorption of iron and vitamin D. These foods contain a range of enzymes, each with its unique role in enhancing food quality, generally through better digestion or enhanced bioavailability. The acidification indirectly activates both the endogenous phytase and the microbial enzymes increasing the mineral bioavailability [9]. 

*Leuconostoc* species are especially considered as good hosts for heterologous protein expression in the food biotechnology industry because they have been identified as safe (GRAS), are not capable of generating endotoxin, grow well under aerobic and anaerobic conditions and secrete certain proteins such as dextransucrase despite being digested by proteolytic enzymes [3]. In this regard, certain leuconostocs have complete genes for the manufacture of certain vitamins and have the potential to improve the nutritional content of fermented foods. *Leuconostoc citreum* and *Leuconostoc mesenteroides* subsp. *mesenteroides* have the entire collection of genes for vitamin B_2_ (riboflavin) production [13].

Furthermore, given their widespread dispersion in the environment and the small number of diseases they produce, it is considered that these bacteria represent very little harm to healthy humans. It is plausible to conclude that *Leuconostoc* is usually safe because of the extensive history of human exposure and ingestion [2]. Moreover, the Food and Drug Administration (FDA) has designated bacteria of the *Leuconostoc* genus as Generally Recognized as Safe (GRAS) due to a history of human exposure and ingestion, while the European Food Safety Authority (EFSA) has granted them the designation of Qualified Presumption of Safety (QPS) [14].

But undoubtedly, one of the most important properties of some *Leuconostoc* ssp. is the capacity to convert fructose to mannitol in sourdough. Therefore, this feature can be exploited as a technical solution to naturally manufacture low-calorie sweeteners and decrease overall sugar content in sweet baked items while maintaining sweetness and flavor. Compared to the other LAB species, *Leuconostoc citreum* and *Leuconostoc mesenteroides* are highly efficient mannitol producers, with yields ranging from 70 to 90% [15]. 

Excess sugar consumption is thought to be a major cause of many disorders, and sweet baked goods are one of the main sources of sugar in human nutrition. Several approaches could be developed to reduce sugar. Currently, there are two well-known strategies: using bulking agents and high-intensity sweeteners to substitute for sugar, or using sweet bulking substances like polyols. Polyols, such as mannitol, are the most commonly utilized sweet bulking agents in the field of sugar reduction. Polyols are sugar alcohols, which are either obtained by chemical or biochemical reduction of sugars or during fermentation by lactic acid bacteria or yeast. Nevertheless, polyols can be used only as a partial replacement for sugar, and the main obstacle in lowering sugar is ensuring product quality in terms of volume, texture, appearance, sweetness and shelf life. Sourdough technology should be regarded as an innovative technique to surmount the negative influence on the quality parameters of sugar-reduced bread goods. Both mannitol and exopolysaccharides production by LAB could contribute to quality improvement. Moreover, not only will consumers benefit from the utilization of sourdough in sugar-reduced products but also, from the point of view of the industry, this strategy provides clean-label products and brings economic benefits [16,17].

The aim of this review is to evaluate *Leuconostoc citreum*’s capacity for mannitol production and its potential application in reducing sugar levels, with a particular focus on sourdough in baked goods and bakery products.

### 1.2. Role in Fermented Plant-Origin Food Products

Several studies have reported on traditionally fermented vegetable-based foods, where *Leuconostoc* ssp., alone or in mixt cultures, are the main microbial agents of fermentation and provide specific sensorial and nutritional properties. *Leuconostoc* is the most common genus of LAB on plants, with *L. mesenteroides* as the most common isolate [18] from squash, peas, cucumbers, greens, cereals, etc. *Leuconostoc citreum* and *Leuconostoc mesenteroides* both contribute considerably to food fermentation. While they have some similarities, important features distinguish them.

Thereby, gundruk, sunki and khalpi are several examples of traditionally fermented foods in Nepal, Sikkim and Bhutan. Gundruk is an indigenous vegetable product from the Himalayas that is fermented, non-salted and has an acidic flavor. It can be enjoyed as both a pickle and a soup and shares similarities with other fermented acidic vegetable products like Korea’s kimchi, Germany’s sauerkraut and Japan’s sunki [19]. Sunki, a traditional fermented food made from radish tap roots, is prepared through pit fermentation, a distinctive method of bio-preserving foods through lactic acid fermentation in the Sikkim Himalayas [9]. Khalpi is a fermented cucumber product made from *Cucumis sativus* L. It is a popular delicacy among the Brahmin Nepalis in Sikkim and stands as the sole known fermented cucumber product in the entire Himalayan region [19]. Kimchi is a traditional Korean fermented vegetable dish prepared from Chinese cabbage, radish, green onion, red pepper powder, garlic, ginger and fermented seafood. The fermentation of kimchi is influenced by temperature; it develops in one week at 15 °C and only takes three days to matures at 25 °C. However, a lower temperature is favorable during kimchi fermentation to avoid the excessive production of strong acid and overripening and to promote an extended period of optimal flavor [20]. The dominant microbial species from kimchi are *Leuconostoc mesenteroides* and *Lactobacillus plantarum*. Various findings suggest that LAB involved in kimchi fermentation encompass a range of species, including *Leuconostoc mesenteroides*, *Leuconostoc citreum*, *L. gasicomitatum*, *Lactobacillus brevis*, *L. curvatus*, *L. sakei*, *L. plantarum*, *L. lactis*, *P. pentosaceus*, *W. confusa* and *W. koreensis* [9]. However, Choi et al., 2003 [21] reported that during the early and mid-stages of kimchi fermentation at 15 °C, *Leuconostoc citreum* was the dominant species when 120 LAB were isolated at random from two different kimchi samples. Moreover, *Leuconostoc citreum* IH 22 played an important role in kimchi fermentation as starter culture, with good capacity to dominate the spontaneous microflora. When this strain was cultivated on 5% sucrose medium, it demonstrated a significantly better tolerance to acid, which resulted in the synthesis of insoluble dextran. The psychrotroph *Leuconostoc citreum* HS-P4 strain was also isolated from kimchi, with outstanding characteristics in terms of growth rate and dextransucrase activity [5]. 

Typically found in the microbial community of plants, *Leuconostoc* spp. have been isolated from sourdough environments on many occasions [22]. Sourdough is another representative fermented product obtained through LAB metabolism, and some strains of *Leuconostoc citreum* can be involved in sourdough fermentation inducing specific transformations. Sourdough is obtained by fermenting a mixture of flour and water, and this process is facilitated by the naturally present or inoculated LAB and/or yeasts, which play a role in its acidification and the leavening characteristics of the dough. LAB enhance the quality of sourdough baked goods by producing metabolites during the fermentation process, which positively influence the products′ texture, flavor and shelf life [23].

Notably, *Leuconostoc citreum* produces mannitol, a low-calorie sugar alternative that is suitable for diabetic and low-sugar meals. Its specialized strains, such as *L. citreum* TR116, have an outstanding ability to reduce sugar content in baked goods such as bread, cookies, buns and cakes [5,6,24,25,26]. Various *Leuconostoc citreum* strains derived from sourdough synthesized dextrans with varying amounts of α-(1-6), α-(1-3) and α-(1-2) linkages, along with mutan and fructans (Amari et al., 2015). These exopolysaccharides (EPSs) increase the viscosity of liquid systems, while in sourdough baked goods, they improve the rheological properties and the crumb structure [24]. These modifications could be explained by some EPS properties such as the water binding capacity and the capacity to combine with proteins in doughs [22]. 

Cells of *Leuconostoc* ssp. can survive in an unfavorable medium for long periods of time. However, the cells could be exposed to stress conditions like pH and temperature variations and oxidative or osmotic changes, and the intensity of these stress factors could lead to cell death or to cell adaptation through suitable chemical reactions in an effort to counteract the adverse effects and recover the capacity for growth or survival. For instance, unfavorable environmental circumstances promote interactions such as the production of glycocalyx in the presence of saccharose and trace minerals, culminating in the formation of a biofilm that defends cells from harmful agents. Evidence has indicated that cell growth ended when internal pH levels of 5.4 to 5.7 were attained, regardless of culture medium composition. The limiting external cellular pH, on the other hand, was heavily impacted by the growth media, namely the kind and concentration of organic acids. On the other hand, low cell lysis was reported at pHs above 6.5 in the presence of Ca^2+^ and Na^+^. Notably, *Leuconostoc* could metabolize citrate when the pH ranged from 6.3 to 4.5, and diacetyl and acetoin were only produced at acidic pHs [3,27]. 

Stress proteins were overexpressed as a reaction of *Leuconostoc mesenteroides* cells to heat shock, while optimal conditions (30 °C) allowed lower pH values, higher citrate utilization and higher ethanol and acetic acid production. However, limiting or excessive contents of nitrogenous compounds could negatively affect *L. mesenteroides′* metabolism.

## 2. Metabolism Characteristics

### 2.1. Metabolism of Sugars in Leuconostoc ssp. and Leuconostoc citreum 

Cogan and Jordan (1994) claimed that because leuconostocs, like other LAB, lack a tricarboxylic acid cycle and a cytochrome system, they cannot receive energy from oxidative phosphorylation. They gain energy instead by substrate-level phosphorylation, which occurs during the fermentation of carbohydrates to lactic acid, ethanol or acetate and CO_2_ [10].

**Figure 1:**
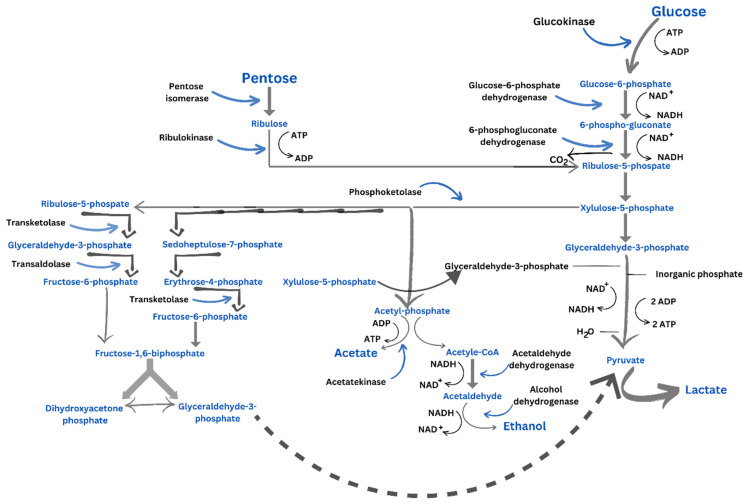
Glucose fermentation pathway. Heterofermentative metabolism (phosphoketolase pathway)—adapted after Teleky et al. 2020 [28].

In the early 1950s, the researchers described the process of glucose fermentation in leuconostocs. The removal of CO_2_ from glucose is followed by the split of the resultant pentose into two-carbon and three-carbon segments, which is facilitated by phosphoketolase. This enzymatic activity produces glyceraldehyde-3-phosphate and acetylphosphate. This mechanism, known as the phosphoketolase route, is seen in heterofermentative lactobacilli as well (Figure 1). The three-carbon section eventually changed to lactate, whereas the two-carbon part converted to ethanol. These end products are critical in replenishing NAD^+^ and sustaining continued fermentation [7]. The overall stoichiometry is as follows:

(1) $$Glucose\to 1\;Lactate+1\;Ethanol+{1\;CO}_{2}+1\;ATP$$

This process is known as the heterofermentative path because it produces compounds other than lactate, as opposed to the homofermentative or glycolytic pathway seen in lactococci, enterococci and several lactobacilli, which only produce lactate:

(2) 1 Glucose=2 Lactate+2 ATP

In the phosphoketase route, one ATP molecule is created for every mole of glucose metabolized, whereas the homofermentative pathway produces two ATP molecules. As a result, in terms of glucose fermentation, the phosphoketolase route is only half as effective as the homofermentative process. Although only the early and terminal enzymes of this route have been thoroughly studied and purified, their fundamental characteristics are well documented [10].

In contrast to glucose fermentation, fructose fermentation produces mannitol and acetate in addition to CO_2_, lactate and ethanol. The fermentation balance is close to the following:

(3) $$3\;Fructose\to 1\;Mannitol+2\;Lactate+0.5\;Acetate+1.5\;Ethanol+{2\;CO}_{2}$$

Mannitol and NAD^+^ are produced in a process catalyzed by mannitol dehydrogenase from fructose and NADH. This process produces more ATP because less acetylphosphate is converted to ethanol to renew the NAD^+^ needed to continue the fermentation. For every mole of acetylphosphate that is not converted to ethanol, two moles of mannitol are generated. Fructokinase and phosphoglucoisomerase are found in leuconostocs fermenting fructose, showing that it is phosphorylated to fructose-6-phosphate before being isomerized to glucose-6-phosphate [10]. When mannitol synthesis is activated, *Leuconostoc citreum* produces acetic acid in order to restore ATP. Acetate is formed from acetylphosphate by an acetate kinase [5,29]. 

Regarding mannitol, it is a low-calorie sweetener and has the potential to replace sucrose, lactose, glucose or fructose in a wide range of food products. It can be metabolized independently of insulin, making it a viable choice for diabetic-friendly foods. *Leuconostoc pseudomesenteroides, Leuconostoc mesenteroides* and *Leuconostoc citreum* are notable for their ability to produce mannitol during the fermentation of fructose [3,6].

*Leuconostoc* ssp. has the capacity to produce exopolysaccharides (EPSs) as the result of environmental stress. EPSs are part of the biofilm layer surrounding the bacterial cells, together with proteins, nucleic acids and lipopolysaccharides. EPSs are categorized as homo- or hetero-polysaccharides, depending on whether they are made up of a single type of monosaccharide building block or several different types. Of the homo-polysaccharides, glucans and fructans are the most often discovered, and both are used as ingredients in the food industry. In the presence of sucrose, *Leuconostoc* ssp. produce α-glucans and β-fructans. The step-by-step addition of glucose moieties to an expanding α-glucan chain is the main mechanism. The glycosidic link in sucrose is first broken during the reaction, creating a covalent glucosyl-enzyme intermediate where the glucosyl is bonded in the donor subsite. The way the acceptor glucan targets the covalent glucosyl-enzyme intermediate dictates the kind of glycosidic bond that is formed [30]. For instance, the primary components of a new water-soluble dextran produced by *Leuconostoc citreum* SK24.002 are α-1,3 and α-1,6 linked d-glucopyranose units at a ratio of 4:5 [31]. In the case of *Leuconostoc citreum* E497 isolated from cereals, the dextran with 11% α-(1→2)-linked branches and 3.5% α-(1→3)-linked branches were produced. Additionally, *Leuconostoc citreum* NM105 may be an excellent prebiotic producer. To further support human health, food formulations may incorporate the natural form of this high-(1–2) branch dextran or the glucooligosaccharides form generated through partial acid hydrolysis. Furthermore, by use of an acceptor reaction with maltose and sucrose, the dextransucrase from *Leuconostoc citreum* NM105 has the potential to be employed in the synthesis of prebiotic oligosaccharides [31]. Moreover, in experimental media with added sucrose and maltose, *Leuconostoc citreum* HJ-P4, HJ-S13 strains were able to produce panose, while dextran synthesis was inhibited. In the meantime, after the isomaltooligosaccharide synthesis occurrence, sucrose releases an equivalent quantity of free fructose, imparting a sweet flavor [5]. It was reported that *Leuconostoc citreum* EFEL2700 demonstrated a typical hetero-type lactic acid fermentation and had the ability to metabolize a wide range of carbohydrates. The genome revealed genes involved in the metabolism of glucose, fructose, sucrose and mannose. Genes for the production of polysaccharides (dextran) from sucrose were also discovered [11].

In wheat flour sourdough supplemented with sucrose, *Leuconostoc citreum* FDR 421 was effective in sucrose conversion into glucose and fructose, while the last one was transformed to mannitol, resulting in the formation of acetic acid. The authors reported a smaller molar ratio of mannitol to acetate (close to 1 compared to 2:1), probably due to the strain’s property of using oxygen as an electron acceptor during the specific conditions of the sourdough propagation, as well as due to the metabolic features of the strain. Fructose was not completely metabolized but the mannitol concentration was positively correlated with the fructose content. As was expected, maltose was also partially metabolized during sourdough fermentation. Sucrose addition facilitate dextran formation. Due to the presence of sucrose and maltose, which dextransucrase uses as an acceptor molecule to create maltooligosaccharides, a low dextran yield was recorded. The formation of maltoologisaccharides can complete dextran production in wheat substrates [22]. *Leuconostoc citreum* TR116 revealed similar behavior regarding glucose, fructose and maltose consumption during wheat sourdough fermentation and a high ability to produce mannitol [29]. *Leuconostoc citreum* DCM65 was also effective in mannitol and acetic acid production in wheat sourdough by fructose consumption [32]. 

When fava beans were inoculated with *Leuconostoc citreum* TR116 to obtain sourdough, glucose, fructose and sucrose were metabolized. Significant amounts of mannitol yielded. Using fructose as a substitute electron acceptor, mannitol dehydrogenase reduced NADH to mannitol. On the other hand, verbascose and stachyose/raffinose in fava bean sourdough showed a general declining tendency during the fermentation process, whereas galactose accumulated. These galactooligosaccharides are known as antinutritional compounds; thus, a little decrease is beneficial. It is also possible that this trend regarding galactooligosaccharides was due to the α-galactosidase activity of fava beans. Concerning EPS production, *Leuconostoc citreum* DSM 5577 revealed glucan synthesis in sucrose-supplemented fava bean sourdough [33].

### 2.2. Citrate Metabolism in Leuconostoc ssp. and Leuconostoc citreum

Leuconostocs like *lactococci* that use citrate as their primary energy source require a fermentable sugar as well. Citrate is metabolized by *Leuconostoc* via the same metabolic mechanism as citrate-positive strains of *L. lactis* [13]. 

**Figure 2:**
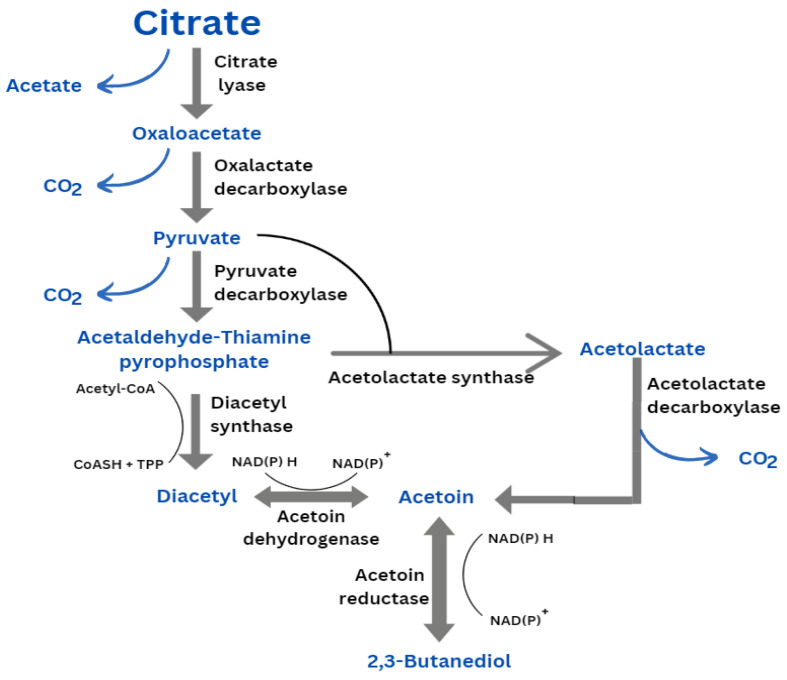
The citrate metabolism pathway in lactic acid bacteria. CoA—coenzyme A; CoASH—free coenzyme A; TPP—thiamine pyrophosphate. Adapted after Cogan and Jordan (1994) [10] and Endo et al. (2021) [13].

The citrate metabolism pathway (Figure 2) includes the following stages: (a) citrate uptake facilitated by citrate permease; (b) citrate splitting into acetate and oxaloacetate via citrate lyase; (c) pyruvate formation by decarboxylating oxaloacetate; (d) pyruvate reduction to lactate. When citrate and sugar are co-metabolized, it supports growth by producing more energy (adenosine triphosphate (ATP)), through the acetate kinase pathway [10,13].

*Leuconostoc* has the ability to convert diacetyl into acetoin and 2,3-butanediol. Notably, citrate could have an important role in sourdough fermentation, providing volatile organic compounds with aromatic properties. Citrate is also metabolized by *Leuconostoc citreum*, which produces volatile chemicals, including acetoin, dyacetil and C4 compounds that contribute to the flavor of fermented products [6,11]. With a distinguished buttery, creamy odor, acetoin is produced by converting citrate to pyruvate, while its production is necessary to equilibrate the redox balance of cell metabolism. Since acetoin is an important aroma that is produced during sourdough fermentation and has a favorable link with wheat bread, larger concentrations of it are more likely to be well liked by customers.

It was reported that *Leuconostoc citreum* TR116 formed diacetyl in barley and oat malt wort during fermentation via acetolactate. But notably, the quantity of asparagine in fermented malt worts was correlated with the diacetyl concentration. Thus, diacetyl could come from oxaloacetate and fumarate, which are both precursors for pyruvate. 2-methylbutanol and 3-methylbutanol were also found in cereal wort fermented by *Leuconostoc citreum* TR116. An increase was reported in these aroma compounds when leucine and isoleucine were present in higher amounts in the wort. Ethylester, an ester with fruity, sweet sensory perceptions was also detected. It is formed when acetate reacts with the end products of ethanol fermentation [26].

## 3. Leuconostoc Citreum and Its Influence in Sourdough

Sourdough fermentation, a traditional biotechnological process, is a venerable practice profoundly rooted in the food business, acting as an early precursor to the revolutionary arrival of yeast fermentation in bread leavening [28,34]. Sourdough with wild lactic acid bacteria and naturally occurring yeasts has long been used to improve the texture, taste and shelf life of bread. This historic method is gaining popularity since it not only imparts specific organoleptic traits but also coincides with contemporary consumer expectations for cleaner, additive-free food items [10]. 

**Figure 3:**
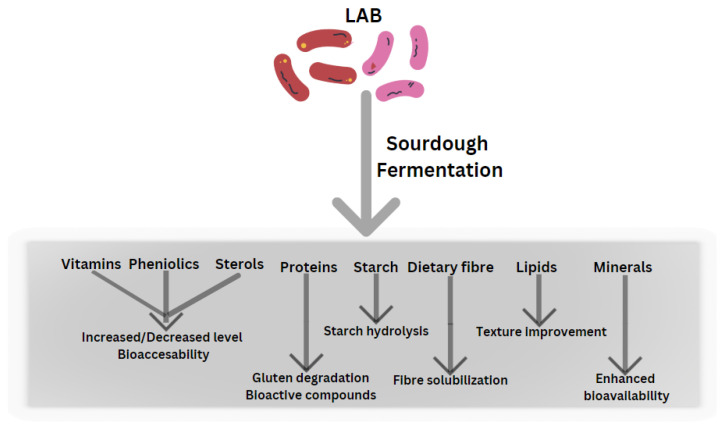
The impact of LAB during sourdough fermentation on nutrient effects. Adapted after Teleky et al., 2020) [38].

The influence of sourdough fermentation on wheat flour-based products is complex, with the acidic environment changing the structure of important dough elements such as arabinoxylans, starch and gluten [28,35]. The nutritional quality is influenced by sourdough fermentation, and this may result in changes to the levels of compounds, as well as the enhancement or inhibition of nutrient bioavailability (Figure 3) [36]. Lactic acid bacteria offers a wide variety of bread quality benefits, ranging from increased shelf life and texture to increased nutritional content and flavor [28,35]. Carbohydrates are absorbed during fermentation, which is vital for the growth of LAB and starch metabolization leading to a reduction in pH, which enhances organic acid production [37].

There is a rising tendency in the food industry to use *Leuconostoc citreum* strains as starter cultures, a phenomenon that has seen a growing presence in documented publications. This increased attention is due to their beneficial metabolic properties, including their capacity to create mannitol, exopolysaccharides (EPS) and other antimicrobial compounds. These advantageous properties have opened the door for the incorporation of *Leuconostoc citreum* strains into a wide range of food items. However, it is worth mentioning that one specific strain, *L. citreum* TR116, has received a lot of attention recently because of its extraordinary potential for functionalizing food and improving its quality and characteristics [7].

*Leuconostoc citreum* TR116 is famous for its distinct capacity to express the mannitol-2-dehydrogenase enzyme, which efficiently catalyzes the transformation of fructose into mannitol, as highlighted by Gänzle in 2015. What truly distinguishes *Leuconostoc citreum* TR116 from its competitors is its exceptional proficiency in consistently achieving a fructose-to-mannitol conversion rate that surpasses 80% across a wide spectrum of food matrices [15]. This exceptional attribute positions TR116 as a suitable choice for the creation of low-sugar food products. Sugar-reduced items are in significant demand due to a rising trend of non-communicable illnesses which include cardiovascular disease, obesity and type 2 diabetes [39]. However, lowering the amount of sugar in food items results in a loss of quality since, in addition to sweetness, sugar also affects texture, structure, microbiological shelf life and flavor [40]. In specific contexts, such as the production of quinoa-based milk alternatives, the utilization of TR116 in a single-strain fermentation process has yielded products enriched with improved nutritional value [41]. These products are characterized by a noteworthy reduction in sugar content, up to 40%, alongside a substantial reduction in glycemic response, a reduction of 35%, as evidenced by the research conducted by Jeske et al. in 2018. 

In a study conducted by Rice et al. in 2020, the impact of *Leuconostoc citreum* TR116 was rigorously examined across various worts, including those derived from barley, oats and wheat. The study found that *Leuconostoc citreum* TR116 not only reduced sugar content but also produced fruity and brandy-like aroma compounds, enhancing the sensory experience. Additionally, *Leuconostoc citreum* TR116 was used in other studies to improve the quality of sugar-reduced sweet baked goods, highlighting its versatility in sugar reduction and flavor enhancement in various food applications [26]. Among the specific aroma compounds formed by this strain are ethylacetate, diacetyl, acetaldehyde, 2–3 butanediol.

In the case of burger buns, a substantial 50% reduction in sugar content was achieved, while retaining characteristics (reduced volume, denser crumb, prolonged shelf life, a brown crust and sweetness) comparable to those of full-sugar burger buns, as demonstrated in the study by Sahin et al. in 2019. The authors discovered that incorporating fermented sourdough with *Leuconostoc citreum* TR116, known for mannitol production, showed promising results in preserving these quality characteristics. Moreover, they suggested that increasing mannitol production could be achieved through continuous-flow sourdough fermentation by introducing more fructose into the system over time [6]. Similarly, Sahin et al. (2019) [42] demonstrated that using sourdough fermented with *Leuconostoc citreum* TR116 in cake formulations resulted in no discernible losses in sensory qualities or techno-functional features despite a 50% drop in sugar content. Furthermore, while focused on low-sugar sourdough cookies with sugar levels as low as 5%, Sahin et al., in 2019 [25], discovered that these goods retained rich flavor, probably due to the incorporation of acids and free amino acids, which supported the Maillard reaction and the subsequent flavor compounds. Beyond its role in sugar reduction, *Leuconostoc citreum* TR116 has been used to improve bread quality, notably through the addition of fermented high-protein fava bean flour, as Hoehnel et al. investigated in 2020 [24]. Moreover, in this specific fermented matrix, the authors report the presence of 15 phenolic compounds with antifungal activity that may contribute to the extension of the shelf life. 

Other strains of *Leuconostoc citreum* were also tested and promising results were obtained. For instance, inoculation of *Leuconostoc citreum* DCM65 in wheat flour resulted in high mannitol production, and a positive correlation between mannitol and acetic acid amounts was also found. Moreover, this strain was able to counterbalance the negative effects of sugar reduction in soft buns due to its capacity to produce both mannitol and EPS [32]. 

Table 1 summarizes the more effective strains of *Leuconostoc citreum* in different sourdough matrices used for sugar-reduced bakery products and baked goods. The main products of fermentation and the effects on the products′ quality characteristics are presented also.

*Leuconostoc citreum* C2.27 isolated from durum wheat semolina was used to obtain liquid sourdoughs and a typical Apulian bread (puccia). Results showed an increased volume and an optimal fermentation quotient compared with *Weissella confusa* C5.7, which was also studied during the experiment. In addition, better textural properties and a softer and more elastic crumb in bread were recorded in the case of *Leuconostoc citreum* C2.27. Notably, when salt was omitted from the bread formulation, any distinct changes in the structural characteristics between the puccia bread made with sourdough and with baker’s yeast were not identified. The bread had a strong flavor and taste even without salt. These results recommend this strain for even yeast-free bread production [43]. 

When *Leuconostoc citreum* FDR241 isolated from rye bran was used for wheat sourdough fermentation, the sourdoughs attained pH values of 4.0 and moderate titratable acidity, exhibiting consistent lactic acid bacterial cell density and acidification characteristics. Throughout the propagation process, the amount of carbohydrates consumed was constant, resulting in the synthesis of mannitol and almost equimolar amounts of lactic and acetic acid. The mannitol amount was correlated with the estimated fructose resulting from back-slopping and sucrose addition. The addition of sucrose facilitated the production of oligosaccharides and dextran, which increased the sourdough’s viscosity by 2 to 2.6 times [22]. 

### Mannitol as Sugar Substitute

In bakery products, naturally produced polyols can contribute to sweetness and flavor, while EPS acts as a bulking agent that enhances dough stability and the texture of the final product [42].

An innovative technical strategy for overcoming quality losses in low-sugar baked goods might be a controlled sourdough fermentation with selected LAB and yeast strains [42]. In a sourdough system, the in situ generation of polyols and exopolysaccharides is required to convert monosaccharides into low-calorie sweetening agents (polyols) and to lower the quantities of monosaccharides by polymerization into long-chain carbohydrates (EPS) [6].

Polyols are also known as “sugar alcohols”, “sugar substitutes” and “hydrogenated carbohydrates”. The name “polyol” comes from the word “polyalcohol” or “polyhydric alcohol”. The suggested names are “polyols” or “hydrogenated carbohydrates”, which specifically emphasize that these compounds are carbohydrates [44]. They are sugar-free sweeteners because they include carbs but no sugars [45]. Polyols are sorbitol, maltitol, lactitol, xylitol, erythritol, mannitol and isomalt. D-mannitol is the most prevalent polyol in nature, due to its production by a variety of species including bacteria, yeasts, fungi, algae, lichens and various plants. It is thought that a French chemist discovered this polyol in the early 19th century [46].
(4)
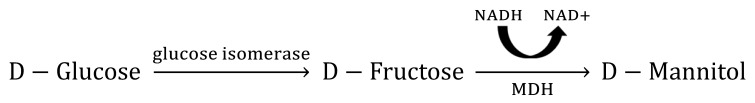


Production of mannitol from glucose in two steps using enzymes originating from hyperthermophilic bacteria (glucose isomerase and mannitol dehydrogenase (MDH)) (image created using BioRender application https://app.biorender.com, accessed on 30 October 2023, redone after Martínez-Miranda et al., 2022) [46].

Polyols, commonly referred to as sugar alcohols, are a family of chemical compounds formed by a multidimensional process that involves either the chemical or biological reduction of sugars or, alternatively, fermentation facilitated by the presence of LAB or yeast [42]. 

In a 2018 study, Sahin et al. discovered that substituting sugar with mannitol in biscuits increased dough hardness when compared to the control. This might cause difficulties in rolling and shaping the dough, influencing the baking process, and perhaps resulting in restricted expansion. Mannitol used as sugar alternative helps to improve dough stability. The solubility of the component influences dough stability. The greater the solubility, the more solvent is present in the solution, resulting in a drop in dough viscosity and poorer stability after heating. Mannitol has a smaller influence on dough viscosity than sucrose or xylitol since its solubility is substantially lower. This increases its stability. As more gas cells remain in the dough, the specific volume of the final product increases. Mannitol, on the other hand, diminishes the flexibility, cohesiveness and chewiness of the cake crumb and adds weak sensory characteristics such as dryness [29].

Because of their bulking qualities and functions, several polyols such as erythritol, xylitol, sorbitol and mannitol, as well as other oligosaccharides, have been developed as alternative sweeteners for food products. Mannitol, in particular, has a wide range of applications outside of the food industry, including medicines, where it is used in the manufacture of chewable tablets and granulated powders. Mannitol is commercially manufactured by reducing fructose or sucrose under high pressure and temperature conditions, with sorbitol created as a by-product [47].

The utilization of mannitol, a sugar substitute, can help address the need for functional ingredients that mimic the rheological characteristics normally offered by sucrose in wheat dough systems. Natural mannitol production by LAB in sourdough is a critical component in the reduction in sugar content for baked goods, allowing for the incorporation of beneficial aspects associated with this sugar substitute. Research on sourdough has indicated an enhancement in bread quality, as discussed by Axel et al. (2015) and Arend et al. (2007) [48,49], and an amplification of flavor and aroma [50]. *Leuconostoc* ssp., among all mannitol-producing LAB, can reduce fructose to mannitol by up to 95% [51].

In general, mannitol can specifically be synthesized from glucose/fructose combinations without producing sorbitol as a by-product, eliminating the need for refined substrates or difficult product purification steps [52]. Under proper oxygen availability circumstances, glucose may be utilized as an energy and carbon source and fructose as an electron acceptor since it can be converted to mannitol by a particular mannitol dehydrogenase. If sugar absorption occurs simultaneously, a bacterium can create up to 2 mol mannitol from 1 mol glucose (and 2 mol fructose). Other metabolic products will include lactic acid, acetic acid and/or ethanol and carbon dioxide [52]. 

## 4. Conclusions

*Leuconostoc citreum* is a lactic acid bacterium that may perform lactic fermentation and operate in sourdough to generate mannitol, a polyol that helps in reducing sugar in baked goods.

This review has highlighted the promising potential of *Leuconostoc citreum* in mannitol production and its significant role in reducing sugar levels, while improving quality, nutritional profiles and sensory qualities, particularly when applied in sourdough for baked goods. Its efficiency in mannitol production, with yields of 70–90%, very good capacity to produce antifungal compounds (acids, ethanol, phenolic compounds, etc.) that could extend shelf life, exopolysaccharide formation with a beneficial role in product texture and specific aroma compounds formation with a positive role for product flavor and taste are among the important features that recommend *Leuconostoc citreum*’s strains as sourdough fermenting agents in some bakery products.

This novel technique fulfills the growing demand for low-sugar baked goods, appealing to health-conscious customers as well as those wanting great taste and quality. 

## Figures and Tables

**Table 1:** Influence of sourdough fermented with different *Leuconostoc citreum* strains on product quality.

Strain	Type of Product, Ingredients	Sourdough Fermentation Parameters	Products of Fermentation	Effects on the Product Quality
*L. citreum* DCM65 [32]	**Soft buns**: flour, tap water, sugar (0, 3, 6 or 9% addition), canola oil, salt, yeast, wheat gluten and sourdough (made of flour, tap water, sucrose, fructose and DCM65)	30 °C/24 h pH = 4	Mannitol—9.8 mg/g EPS—dextran Acetic acid—2.5 mg/g Lactic acid—3.1 mg/g	- Increased shelf life (visible molds only after 12–16 days) - Smaller slice area and finer pore structure than the reference buns - Higher pore cells and positive correlation with mannitol and acetic acid - Smaller volume of *L. citreum* DCM65 buns compared to reference buns, in inverse relationship with mannitol and acetic acid contents - EPS decreased the crumb firmness
*L. citreum* TR116 [6]	**Burger buns**: wheat flour, yeast, sucrose, salt, wheat gluten, wheat starch, ascorbic acid, sunflower oil, sodium stearoyl lactate, tap water and sourdough (made of: SD1: 50% flour, 50% tap water; SD2: 40% flour, 50% tap water, 10% fructose)	30 °C/48 h pH = 4.2	Maltose ~ * 4.95 g/100 g DM (SD1) ~2.62 g/100 g DM (SD2) Mannitol Acetic acid ~2.62 g/100 g DM (SD1) ~0.52 g/100 g DM (SD2) Lactic acid ~2.87 g/100 g DM (SD1) ~1.70 g/100 g DM (SD2)	- Specific volume, crumb hardness and chewiness revealed no significant differences between sugar-reduced sourdough burger buns and the control - No significant differences in sweetness and sourness between sugar-reduced buns and control
*L. citreum* TR116 [25]	**Biscuits**: flour with protein content of 8% and a moisture of 14%, sucrose, shortening, sodium stearoyl lactylate, salt, baking powder and sterile tap water (SD: 50% flour, 50% tap water; SD_FRU_: 40% flour, 10% fructose, 50% tap water; SD_FS_: 40% flour, 5% fructose, 5% sucrose, 50% tap water)	30 °C/30 h pH 5.41–5.47	Mannitol ~0.04 g/100 g (SD) ~0.48 g/100 g (SD_FRU_) ~0.41 g/100 g (SD_FS)_ EPS ~0.71 g/kg (SD_FS_) Maltose Acetic acid ~0.204 g/100 g (SD_FS_) ~0.195 g/100 g (SD_FRU_) Lactic acid ~0.112 g/100 g (SD) ~0.076 g/100 g (SD_FRU_, SD_FS_)	- Improvement of the the viscoelastic properties, dough stickiness, hardness biscuit firmness improvement - Increased sweetness and flavor intensity - Improved biscuit color
*L. citreum* TR116 [42]	**Cakes**: flour, sterile tap water, sugar, canola oil, salt, yeast, wheat gluten, sourdough (SD: 50% sterile tap water, 50% flour;SD_FRU_: 50% sterile tap water, 40% flour, 10% fructose)	30 °C/30 h	Mannitol ~0.70 g/100 g DM Acetic acid Lactic acid	- Improved the pasting properties - Similar specific volume to the full-sugar control - Significantly softer crumb - Increased sweetness perception (+93%), aroma (+30%) and flavor (+25.5%)
*L. citreum C2.27* [43]	**Bread**: wheat flour, sterile tap water and liquid sourdough (made of wheat flour, sterile tap water and bacterial suspension)	30 °C/16 h pH 4.48	Acetic acid ~4.30 mmol/kg Lactic acid ~15.52 mmol/kg	- Increased specific volume - Decreased resilience and fracturability - Lower hardness of bread - Lighter crust
*L. citreum FDR241* [22]	**Bread sourdough**: wheat flour, water, sucrose	30 °C/24 h pH 4	Mannitol ~2.15 mmol/kg Maltose ~3.70% Fructose ~1.73% Glucose ~0.18% Acetic acid 1.65% Lactic acid 3.38% EPS—dextran ~0.71%	- EPS improved structure and volume - Increased softness - Delay in staling
*L. citreum* TR116 [24]	**Bread**: Wheat flour, fava bean ingredients (dehulled flour, DF, and high-protein flour, PR), baker’s yeast, NaCl, oil, water	30 °C/24 h pH 5.47	Mannitol ~0.63–0.77% DM Raffinose ~0.84–0.93% DM Verbascose ~0.49–0.54% DM Galactose ~0.75–0.99 % DM Acetic acid ~0.84–1.14 % DM Lactic acid ~1.23–1.33% DM	- Improved gluten quality - Increased volume of bread - Decreased crumb hardness - Crumb structure with a slight tendency towards bigger cells - Lower crust lightness

*—Approximately; DM—dry matter; SD1—first type of sourdough; SD2—second type of sourdough; SD—sourdough; SD_FRU_—sourdough with fructose; SD_FS_—sourdough with fructose and sucrose.

## Data Availability

No new data were created or analyzed in this study.
